# Supplementary figures and images for: HIV-1 cell-to-cell spread overcomes the virus entry block of non-macrophage-tropic strains in macrophages
Source: PLoS Pathog. 2022 May 27;18(5):e1010335. doi: 10.1371/journal.ppat.1010335 (PMC9182568; doi:10.1371/journal.ppat.1010335)

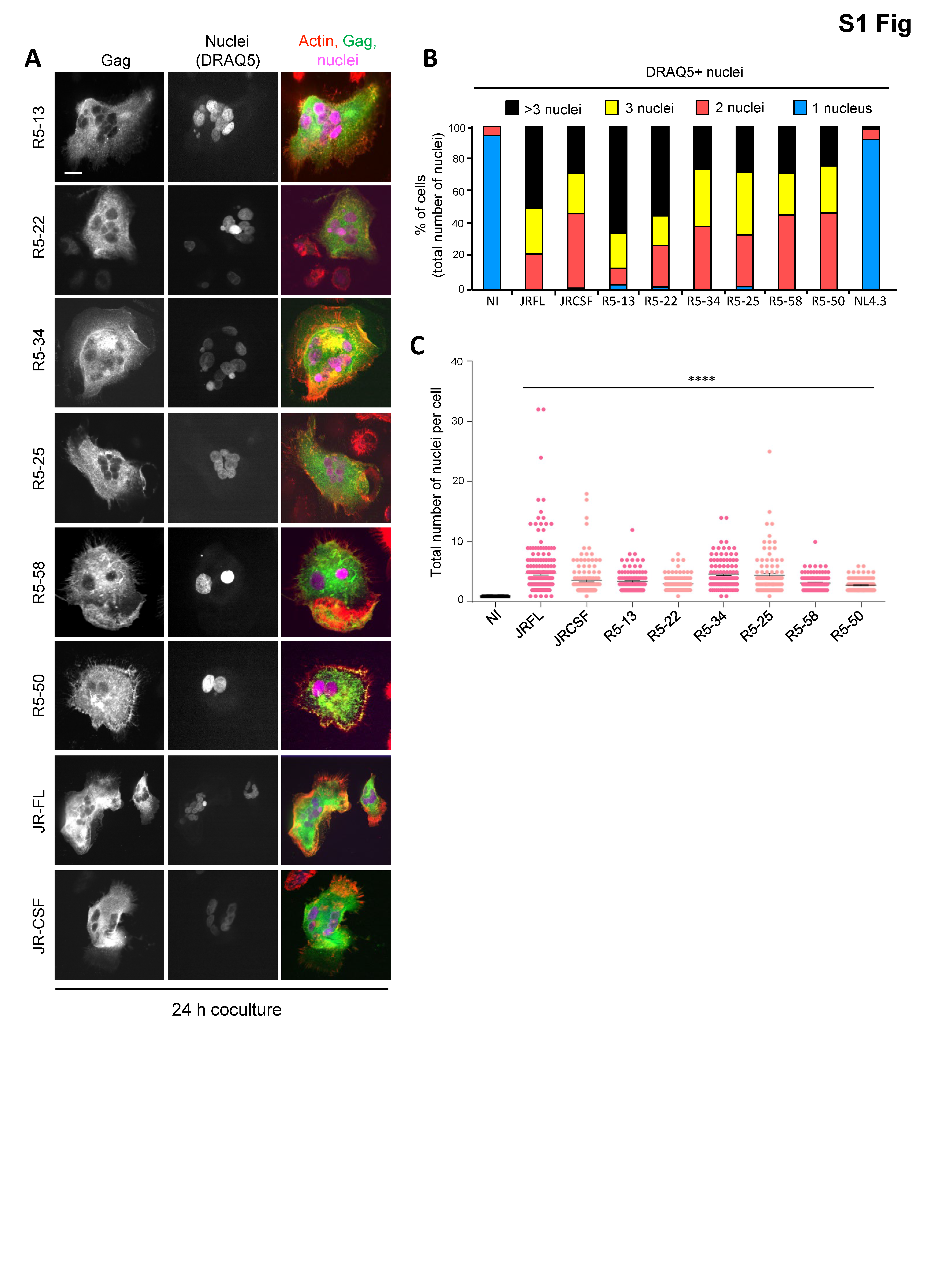

Supplement: S1 Fig — Jurkat cells infected with the indicated virus clones were co-cultured for 24 h with MDMs. After elimination of Jurkat cells, MDMs were stained with anti-Gag, phalloidin (actin) and DRAQ5 (nuclei), and analyzed by confocal microscopy (scale bar, 10 μm). Representative images are shown in A). The total number of nuclei (DRAQ5+) per Gag+ MDM was quantified from images on at least 200 cells. MDMs cocultured with non-infected Jurkat cells were used as negative controls (NI). In B), results are expressed as the percentages of Gag+ MDMs with 1, 2, 3 or more than 3 DRAQ5(+) nuclei. In C), results are expressed as the number of DRAQ5(+) nuclei per Gag+ MDM; each dot corresponds to 1 cell. Horizontal bars represent means +/- 1 SEM. The results shown are representative of at least 6 independent experiments performed with MDMs from at least 6 different donors. (TIF) [file ppat.1010335.s001.tif]

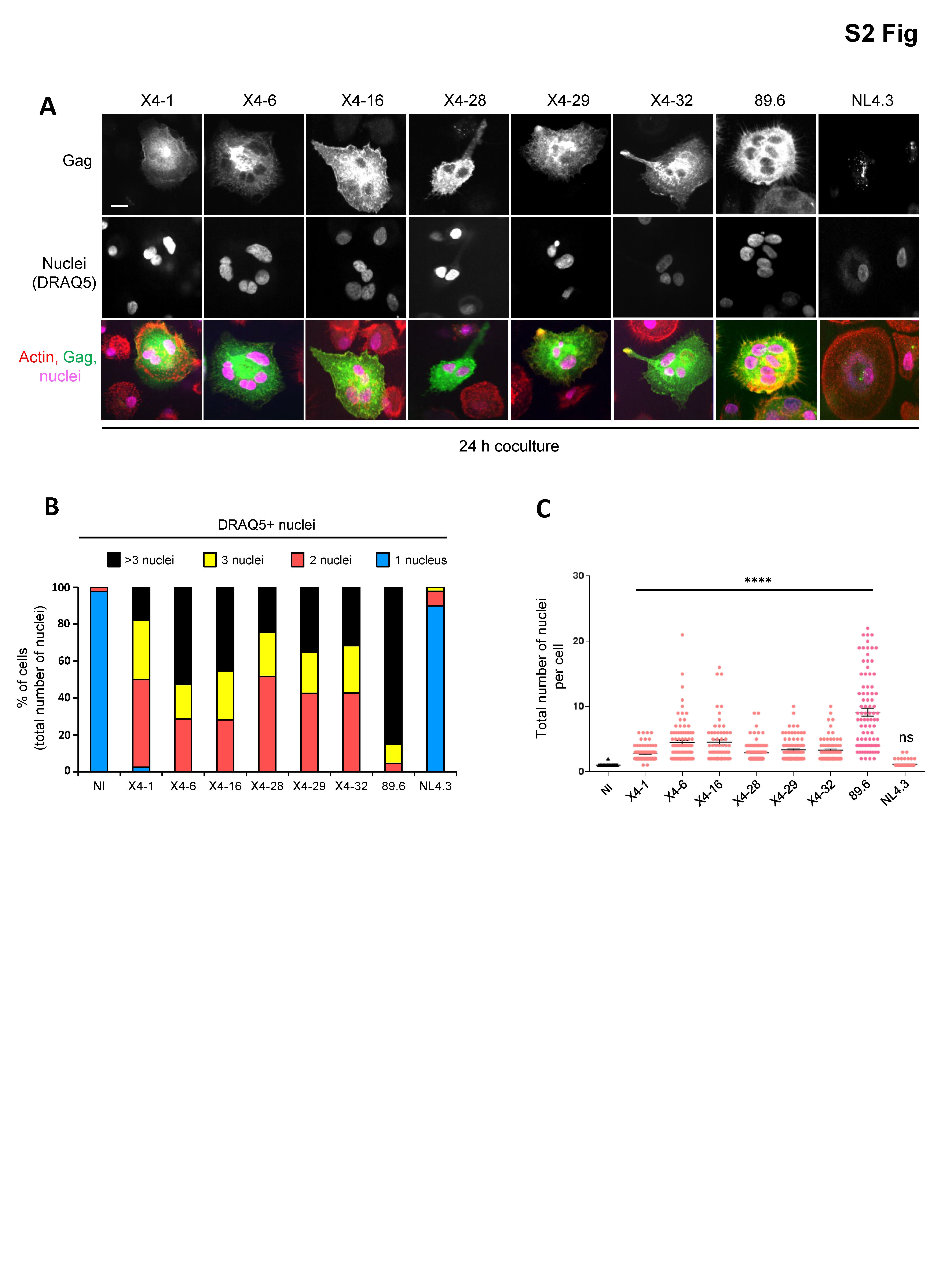

Supplement: S2 Fig — Jurkat cells infected with the indicated viruses were co-cultured for 24 h with MDMs. After elimination of Jurkat cells, MDMs were stained with anti-Gag, phalloidin (Actin) and DRAQ5 (nuclei), and analyzed by confocal microscopy (scale bar, 10 μm). Representative images are shown in A). The total number of nuclei (DRAQ5+) per Gag+ MDM was quantified from images on at least 100 cells. MDMs cocultured with non-infected Jurkat cells were used as negative controls (NI). In B), results are expressed as the percentages of Gag+ MDMs with 1, 2, 3 or more than 3 DRAQ5(+) nuclei. In C), results are expressed as the number of DRAQ5(+) nuclei per Gag+ MDM; each dot corresponds to 1 cell. Horizontal bars represent means +/- 1 SEM. The results shown are representative of at least 6 independent experiments performed with MDMs from at least 6 different donors. (TIF) [file ppat.1010335.s002.tif]

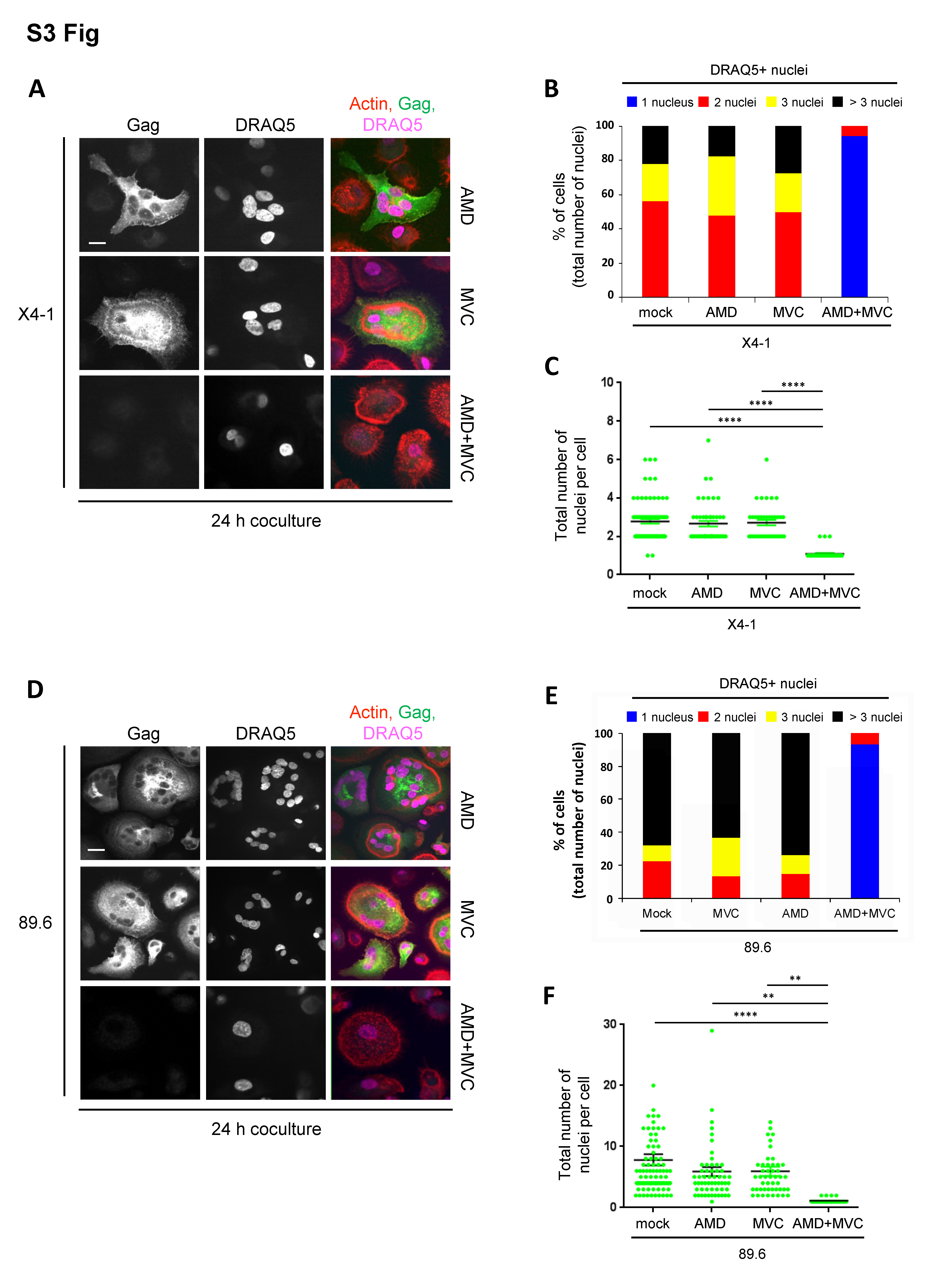

Supplement: S3 Fig — Jurkat cells were infected with X4-1 (A-C) or 89.6 (D-F) Env-pseudotyped viral clones, and then cocultured for 24 h with MDMs pretreated or not (mock) with AMD3100, MVC or both. After elimination of Jurkat cells, MDMs were stained with anti-Gag, phalloidin (Actin) and DRAQ5, and analyzed by confocal microscopy (scale bar, 10 μm). Representative images are shown in A) and D). The total number of nuclei (DRAQ5+) per Gag+ MDM was quantified from images on at least 100 cells. MDMs cocultured with non-infected Jurkat cells were used as negative controls (NI). In B) and E), results are expressed as the percentages of Gag+ MDMs with 1, 2, 3 or more than 3 DRAQ5(+) nuclei. In C) and F), results are expressed as the number of DRAQ5(+) nuclei per Gag+ MDM cocultured with infected Jurkat cells; each dot corresponds to 1 cell. Horizontal bars represent means +/- 1 SEM, and statistical significance was determined with the Mann-Whitney U-test (**, P < 0.01; ****, P < 0.0001). The results shown are representative of at least 4 independent experiments performed with MDMs from at least 4 different donors. (TIF) [file ppat.1010335.s003.tif]

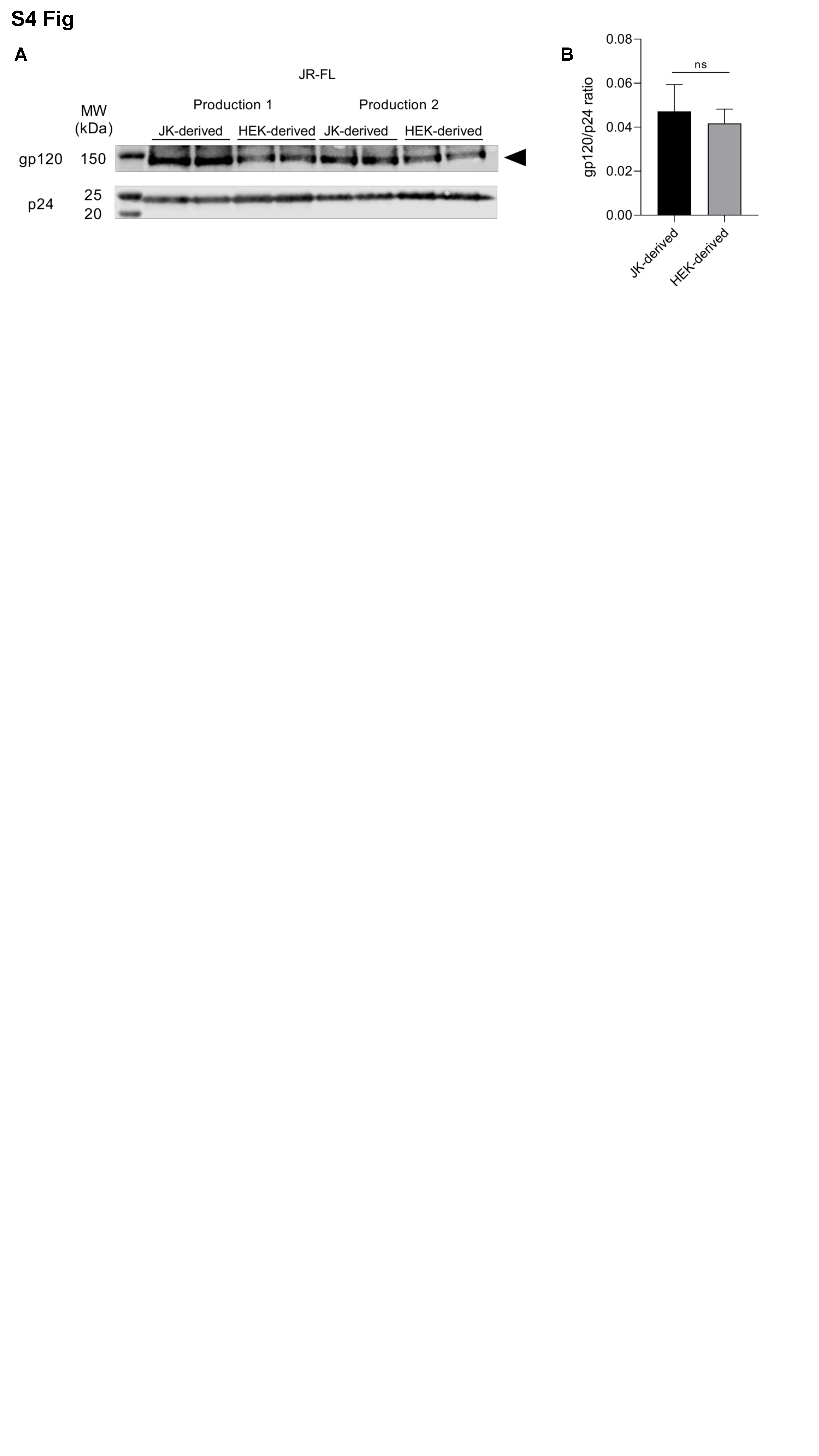

Supplement: S4 Fig — A) Western blot analysis of gp120 and p24 expression into viruses, Jurkat or HEK 293T-derived, pseudotyped with JR-FL Env. For each virus, 130 ng of Gag p24 were solubilized in lysis buffer containing XT sample buffer (Biorad), Invitrogen NuPAGE sample reducing agent and 1% Triton X-100, incubated for 5 min at 70°C, loaded onto Biorad Criterion XT 4–12% Bis-Tris gels under reducing conditions and then transferred to nitrocellulose membrane. Membranes were blocked with Odyssey blocking buffer (Li-COR) (for p24 detection) or TBS containing 5% BSA and 0.05% NaN3 (for gp120 detection) and then incubated overnight at 4°C with a sheep anti-HIV-1 gp120 polyclonal antibody (clone D7324, Aalto Bio Reagents) or for 1 h at RT with a mouse anti-HIV-1 p24 mAb (clone 749140, R&D Systems). Membranes were incubated with the following species-specific secondary antibodies: DyLight 800-conjugated donkey Anti-Sheep IgG (Novusbio) and IRDye 800CW-conjugated goat Anti-Mouse (Li-COR) (dilution: 1/10,000). Signals were detected with a Li-COR Odyssey scanner and quantified using ImageStudioLite software. Arrow indicates gp120 bands. Results from two independent experiments with two distinct virus productions, carried out in duplicate, are shown. B) Band intensity ratios (means ± SD) of gp120 to p24. Statistics: Mann-Whitney U-test. (TIF) [file ppat.1010335.s004.tif]

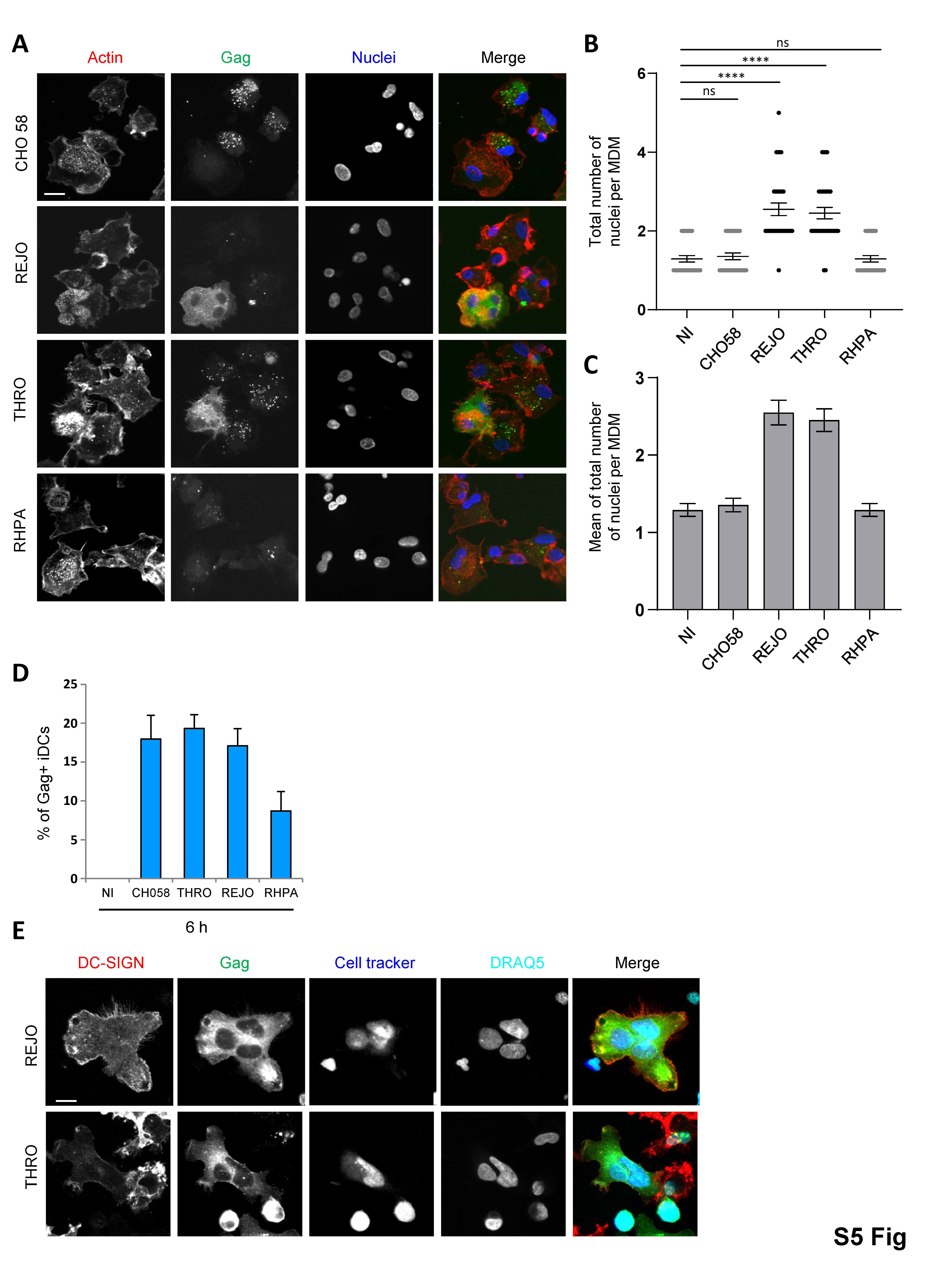

Supplement: S5 Fig — A) Blood primary CD4+ T cells were purified, infected with the indicated T/F viruses, and then cocultured with autologous MDMs for 24 h. MDMs were then fixed and stained with phalloidin (Actin), anti-Gag and Dapi (Nuclei), and analyzed by confocal microscopy. Representative images are shown. B) and C) The total number of nuclei (Dapi+) per Gag+ MDM was quantified from images on at least 30 cells. MDMs cocultured with non-infected CD4+ T cells were used as negative controls (NI). In B), results are expressed as the number of Dapi(+) nuclei per Gag+ MDM; each dot corresponds to 1 cell. Statistical significance was determined with the Mann-Whitney U-test (ns, not significant, P > 0.05; ****, P < 0.0001). In C), results are expressed as the means of total nucleus number (Dapi+) per Gag+ MDM. The results represent means of 2 independent experiments performed with MDMs of 2 different donors. Horizontal bars represent means +/- 1 SEM. D) Blood monocytes were differentiated into immature DCs for 5 days with GM-CSF and IL-4 and then cocultured for 6 h with Jurkat cells infected with the indicated T/F viruses. The percentage of CD11c+/Gag+ DCs was determined by flow cytometry after intracellular Gag staining. DCs cocultured with non-infected Jurkat cells were used as negative controls (NI). E), Jurkat cells infected with THRO and REJO viruses and prelabeled with CellTracker were cocultured for 6 h with DCs. DCs were then stained with anti-Gag, anti-DC-SIGN and DRAQ5 (nuclei), and analyzed by confocal microscopy (scale bar, 10 μm). (TIF) [file ppat.1010335.s005.tif]
