# Supplementary material for: HIV-1 cell-to-cell spread overcomes the virus entry block of non-macrophage-tropic strains in macrophages
Source: PLoS Pathog. 2022 May 27;18(5):e1010335. doi: 10.1371/journal.ppat.1010335 (PMC9182568; doi:10.1371/journal.ppat.1010335)
Supplement: Attachment — Submitted filename: Response letter to the reviewers comments.docx [file ppat.1010335.s006.docx]

**Response letter to the reviewers’ comments (PPATHOGENS-D-22-00224)**

We thank the Editor and the Reviewers for having reviewed our work, for their constructive comments and for the positive assessment of the quality of our manuscript.

- **Reviewer #1**

**“I support publication, perhaps the following minor aspects could be clarified:**

**- Line 117: 293- affinofile? Meaning?”**

The 293-Affinofile cell line is a dually inducible cell line developed by the Benhur Lee’s laboratory in which the CD4 and CCR5 expression levels can be simultaneously and independently manipulated (initially described in Johnston et al., 2009, J. Virol.). This cell line has been widely used to study the dependence on receptor expression levels for cell-free HIV-1 entry (see for instance our previous publications: Garcia-Perez, Retrovirology, 2015; Colin, PLoS Pathog., 2018). Under conditions where CD4 cell surface expression is low, as in macrophages, these cells have been largely used to discriminate between M-tropic and non-M-tropic viruses, and generally the results paralleled those obtained using MDMs as target cells. In our manuscript, references #22 and #23 are representative examples of this particular use of Affinofile cells. These different points have been now highlighted in the revised manuscript (lines 117-121 of the revised version).

**“Line 160: Are used NL43 reporter viruses nef defect and how does this affect viral infectivity and readout?”**

Indeed, the pseudotyped viruses used in this study are viruses in which the *nef* gene has been replaced by the *Renilla luciferase* gene, as described in detail in our previous work (Colin, PLoS Pathog., 2018). Many previous studies, including ours (Madrid, 2005, J. Biol. Chem; Laguette, 2009, J. Virol., Bouchet, 2011, Blood; Bouchet, 2012, J. Virol.) clearly showed that Nef positively influences virus infectivity and replication, through downregulation of CD4 and inhibition of SERINC5 restriction, while Nef does not directly play a role in virus binding to CD4 and coreceptor and virus entry. Of note, most of these studies were performed using human lymphocytic CD4+ cell-lines or primary CD4+ T cells as target cells. In contrast, our previous published data indicate that infectivity of cell-free Nef-deleted viruses is not affected in MDMs and is similar to that measured with wild-type viruses (Verollet, 2015, Blood).

Finally, we have included data in the figure below showing that wild-type and Nef-deleted viruses were similarly transferred from infected CD4+ T cells to MDMs (panel A) through cell-cell fusion (panels C and D) leading to equivalent virus production 4 and 6 days after cell-to-cell transfer (panel B). Therefore, these data indicate that the use of viruses lacking Nef expression does not significantly affect virus spreading in multinucleated giant cells (MGCs) formed after initial cell-to-cell transfer from infected CD4+ T cells. Altogether, these findings confirm that Nef has no impact on cell-free or cell-to-cell virus entry and spreading in macrophages and do not change our conclusion that M- and non-M-tropic viruses can effectively enter macrophages through cell-to-cell transfer from infected CD4+ T cells.

**Cell-to-cell transfer of Nef-deleted HIV-1 from infected T cells to MDMs.** Jurkat cells were infected with the wild-type or Nef-deleted NLAD8 HIV-1 strains (NLAD8_WT and NLAD8_ΔNEF, respectively), and then cocultured for 24 h with MDMs. After elimination of Jurkat cells, MDMs were analyzed by flow cytometry after staining with anti-Gag antibodies (**A**), immunofluorescence and confocal microscopy after staining with anti-Gag, phalloidin (Actin) and Dapi (Nuclei) (**C** and **D**), or for p24 virus production 4 and 6 days after coculture (**B**). In **A**), results are expressed as the percentage of Gag+ MDMs related to the NLAD8_WT strain and are the means of 3 independent experiments performed with MDMs of 3 different donors. In **B**), results are expressed as the concentration (ng/mL) of p24 measured by ELISA in the cell-culture supernatant. In **C**), representative confocal images are shown. In **D**), the total number of nuclei (Dapi+) per MDM was quantified from images on at least 50 cells, and the results are expressed as the number of nuclei per cell; each dot corresponds to 1 cell. MDMs cocultured with non-infected Jurkat cells were used as negative controls (NI). Horizontal bars represent means +/- 1 SEM. Statistical significance was determined using the Mann-Whitney U-test (ns, non-significant, P>0.05).

**« Fig. 1A/B. Do results correlate (infectivity in CD4TL vs macrophages)? Same for 1C/D and also (as control) 1A with 1C or 1B with 1D**

**line 365 obtained »**

It can be inferred from **Fig. 1** that there is no correlation between virus infectivity in CD4TL (**panels A** and **C**) and virus infectivity in (**panel B**) or fusion with (**panel D**) MDMs. To help the reader, this is now more clearly indicated in the revised manuscript (on page 8). For instance, virus R5-34 was one of the viruses with the lowest infectivity in CD4TL (**panels A** and **C**), whereas it was the most effective at infecting and fusing with MDMs (**panels B and D**). Conversely, other viruses that showed high infectivity in CD4TLs did not infect MDMs at all (*e.g.* JR-CSF, R5-50, X4-1). **Fig. 1** also shows that overall, infectivity in CD4TL varied similarly between viruses regardless they contain BLaM-vpr (**C**) or not (**A**) (for instance, JR-FL and R5-34 were the most and least infectious viruses in CD4TL in their versions with or without BLaM-vpr). Finally, we have now more clearly indicated in the revised manuscript that there is a correlation between infectivity in MDMs (**Fig. 1B**) and the capacity of viruses to fuse with these cells (**D**) (page 9 of the revised manuscript). In other words, the viruses that are able to fuse with MDMs are those for which we have found that they are able to infect these cells.

**“Authors discussed several mechanism for why cell-cell transfer is more efficient than cell free infection. Authors should also consider that an increased concentration of virions produced by the donor cell may also enhance infection. This could be mimicked in a cell-free infection experiment by adding concentrated virus in a very small volume to macrophages followed by a spinoculation. Would this allow infection rates comparable to those via cell-cell transfer?”**

As shown in **Figs 5** and **6** of the manuscript, we have been able to find conditions, for the M-tropic strain JR-FL, leading to similar levels of infected macrophages by cell-free infection and through viral cell-to-cell transfer from infected Jurkat cells. High levels of infection by the cell-free mode could be achieved using high amounts of viral particles (450 ng/mL of p24 Gag, thus two logs higher than the concentration used in the experiments shown in **Fig. 1**). However, for non-M-tropic viruses, we were not able to obtain the same results, even using huge amounts of p24 Gag. The levels of infection of macrophages by the cell-free mode consistently remained dramatically lower for non-M-tropic viruses compared to M-tropic viruses (**Figs 2 and 3**). This is also illustrated in the attached Figure below, which we have just generated to test the assumptions of the reviewer. Even at high virus concentration and using a spinoculation protocol, cell-free infection of macrophages with non-M-tropic JR-CSF remained much lower than that of JR-FL, whereas similar infection levels could be obtained for JR-FL and JR-CSF when they are transferred from T cells (**Fig 6A**).

**Infectivity of the M- and non-M-tropic virus strains JR-FL and JR-CSF in MDMs.** The indicated concentrations of viruses were spinoculated (60 min, 2000 g at 4°C) onto MDMs seeded in flat bottom 96-well plates. Then, MDMs were further incubated at 37°C for 48 h. Infectivities were determined by measuring Renilla luciferase activity in the cell lysates. One experiment out of two performed in triplicate is shown. Error bars : SEM.

These findings strongly suggest that an increased concentration of viruses produced by the donor T cell is not the reason why infection of macrophages by cell-to-cell viral transfer is more efficient than cell-free infection and allows non-M-tropic viruses to infect macrophages. Therefore, this contrasts to what has been proposed for the viral transfer through the virological synapse (e.g. Duncan, 2014, J. Virol.). Actually, our fluorescence microscopy analysis shows that most of infection events of macrophages during the coculture with infected CD4 T cells result from cell-cell fusion, as revealed by the presence of at least one T cell nucleus in almost all infected macrophages (**Figs 2, 3** and **7**), and not from infection by cell-free viruses produced by the donor T cell. In the revised manuscript, we explain this by the fact that viruses that are produced from T cells have low infectivity in MDMs (**Fig 4**), even though we confirmed that they are present at high concentrations in the coculture medium (our results here, and Bracq, 2017, J. Virol.).

- **Reviewer #2**

**“• The technical detail that Jurkat cells were initially infected with VSV-g pseudotyped viruses should be specified in the Results and not only in the Method section.”**

As requested by the reviewer, the information that Jurkat cells were initially infected with VSVg-pseudotyped viruses co-expressing HIV-1 Env as well as VSVg is now specified in the “Results” section of the revised manuscript (page 10, lines 220-224).

**“• In the co-culture experiments, “Jurkat cells were removed by extensive washes” (line 217). I assume that this protocol implies that MDM were adherent to the plastic surface while Jurkat cells were in suspension. This (or an alternative explanation) should be specified in the Results.”**

The text has been modified in the “Results” section as recommended by the reviewer (page 10, lines 225-226 of the revised manuscript), indicating that Jurkat cells in suspension were eliminated by extensive washes, and the adherent MDMs were then harvested, stained for the intracellular viral Gag p24 antigen before flow cytometry analysis. In addition, we have previously shown in Bracq, 2017 (J. Virol.) that this extensive washing protocol led to elimination of more that 98% of T cells.

**“Minor points**

**• The sentence “Therefore, M-tropic viruses should be viewed as more likely to infect MDMs as compared to non-M-tropic viruses” (lines 179-181) is a tautology and should be omitted.”**

As requested, this sentence has been removed from the “Results” section (lines 184-186, pages 8 and 9 of the revised manuscript).

**“• In relationship to the hypothesis described in lines 307-308, the paper by Massari et al. (“In vivo T lymphocyte origin of macrophage-tropic strains of HIV. Role of monocytes during in vitro isolation and in vivo infection” by F. Massari et al., J Immunol, 1990 Jun 15;144(12):4628-32) should cited.”**

As suggested by the reviewer, we have now referenced this work in the reference list (Ref # 68 of the revised manuscript). This study in the context of our work is commented in the “Discussion” section on page 21 (lines 490-493) of the revised manuscript.

**“• Concerning the experimental results described in lines 332-340, an appropriate control would have been to incubate HEK cells with Jurkat-derived supernatant to see whether some inhibitory activity could be detected.”**

As shown in **Fig 4D**, purification and washing of virus particles that are produced from Jurkat cells do not restore their infectivity in MDMs, ruling out the possibility that the decreased infectivity compared to that of HEK cell-derived viruses is due to the presence of inhibitory molecules in the cell-culture supernatant of Jurkat infected cells. In light of the reviewer’s comments, we also tested whether incubation of MDMs with Jurkat cell culture supernatant could influence their permissiveness to HIV-1 (JR-FL). However, this was not the case, confirming that there is no inhibitory activity in the supernatant of Jurkat cells (NB/ similar results were found in the presence of the supernatant of HEK cells), in agreement with the results shown in the manuscript.

**“In addition, viruses generated from primary T cells, rather than a cell line, should have been tested for their capacity to infect MDM. Perhaps the author could consider these observations expanding the discussion of the results.”**

The experiment proposed by the reviewer has in fact already been performed and is presented in **Fig 4C**. This experiment illustrates that similarly to Jurkat cell-derived viruses, JR-FL generated from primary CD4+ T lymphocytes has a reduced ability to infect macrophages, compared to when it is produced from HEK cells (see **Fig. 1** for comparison).

- **Reviewer #3**

**1) The study exclusively uses the Jurkat T cell line as the "donor" in the T cell- macrophage infections. It seems important to confirm the mechanism using primary CD4-positive T cells, perhaps using the two transmitted/founder viruses that show it, to maximize the relevance.**

As suggested by the reviewer, the cell-to-cell virus transfer of the 4 transmitted/founder (T/F) viruses to macrophages has been now analyzed using purified primary blood CD4+ T cells as virus-donor cells and autologous MDMs as target cells (new **Fig 7C**, and new **S5A**, **S5B** and **S5C** **Fig**). As now shown in new **Fig 7C**, the 4 T/F viruses were efficiently transferred to MDMs when primary CD4+ T cells were used as virus-donor T cells (black bars)**,** whereas only low level of virus transfer was detected when MDMs were infected with cell-free viruses (**Fig 7C**, red bars), confirming that virus cell-to-cell transfer is more efficient than cell-free infection. In addition, immunofluorescence analyses performed using either infected Jurkat or primary CD4+ T cells show that only THRO and REJO T/F viruses were transferred to MDMs by cell-cell fusion with virus-donor Jurkat or primary CD4+ T cells, whereas the 2 other T/F viruses, CHO58 and RHPA, were captured by MDMs but without cell-fusion with infected T cells (**S5 Fig**).

**2) Two of the transmitted/founder viruses (CHO58 and RPHA) that score positively in the FACS assay for Gag after cell-cell infection seem microscopically to have only been taken up as virus particles (puncta vs. the diffuse staining consistent with synthesis of new Gag protein) and do not seem to have induced multinucleated cells or cells that contain T cell nuclei. The authors should clarify that that is not likely to represent productive infection. They could choose to document this by continuing the experiment for several days; presumably viral spread in the culture would not be evident for these.**

In another manuscript we have recently submitted (Mascarau et al.) for publication regarding characterization of the different mechanisms that might be involved in macrophage infection by virus cell-to-cell transfer from infected CD4+ T cells, we were able to detect, in addition to the multinucleated Gag macrophages, a few percentage of mononucleated Gag+ MDMs (less than 10%) with a faint and dotted Gag signal outside of the acidic endocytic compartments reminiscent of the images shown in **Fig 7D** and **S5A Fig** of the present manuscript regarding CHO58 and RHPA T/F viruses. We show in this submitted manuscript by Mascarau et al. that only the cell-cell fusion mode of virus transfer leads, as suggested by the reviewer, to a high productive infection evidenced by level of p24 measured in the cell-culture supernatant several days after the initial coculture (in agreement with our previous publications by Bracq et al, 2017, J. Virol., and Xie et al., 2019, mBio). However, these experiments have been mainly done using the NLAD8 R5 macrophage-tropic HIV-1 strain, and we cannot formally exclude that some T/F viruses transferred through this synapse-like mode of virus capture could lead to a weaker productive infection of the macrophage targets. We are now starting to explore in details this important specific point regarding the different mechanisms of cell-to-cell transfer and spreading in macrophages used by T/F viruses.

**3) Figure 7 panel H: Are the label for AMD and MVC incorrect (switched)? It appears that AMD is inhibiting formation of multinucleated cells rather than MVC, which is not as stated in the text of the Results and would not make sense.**

As mentioned by the reviewer, this mistake is now corrected on **Fig 7** (panel I of the new figure), and green bars correspond to AMD treatment while light blue bars correspond to MVC treatment.

**4) Legend of Figure 3 could restate the meaning of the grey bar in panels A and B (presumably same as in Figure 2).**

In **Fig 2**, the grey bars correspond to the viruses pseudotyped with R5 M-tropic Envs as defined from the data reported in **Fig 1**. In **panels A and B** of **Fig 3**, the grey bar corresponds to the virus pseudotyped with Env from the dual(R5X4)-tropic isolate 89.6, which was used as a positive control in these virus cell-to-cell transfer experiments. This point was now indicated in the revised version of the **Fig 3** legend.

**“5) Line 360: "inverse agonist" Is "antagonist" meant?”**

The term “inverse agonist” refers to the capacity of maraviroc to stabilize CCR5 in a conformation, which is uncoupled from G proteins (i.e. the opposite of what agonists do) (our previous work by Garcia-Perez, J. Biol. Chem., 2011, among others from other labs). From a pharmacological standpoint, antagonists differ from inverse agonists in that they do not induce conformational changes of the receptor. To avoid confusion, we have replaced the term “CCR5 inverse agonist” by the term “CCR5 entry inhibitor” in the revised version of the manuscript (line 369).
